# Human-Specific Endogenous Retroviruses

**DOI:** 10.1100/tsw.2007.270

**Published:** 2007-11-26

**Authors:** Anton Buzdin

**Affiliations:** Shemyakin-Ovchinnikov Institute of Bioorganic Chemistry, Moscow 117871, Russia

**Keywords:** Human specific, retroelements, genome, endogenous retroviruses, human evolution

## Abstract

This review focuses on a small family of human-specific genomic repetitive elements, presented by 134 members that shaped ~330 kb of the human DNA. Although modest in terms of its copy number, this group appeared to modify the human genome activity by endogenizing ~50 functional copies of viral genes that may have important implications in the immune response, cancer progression, and antiretroviral host defense. A total of 134 potential promoters and enhancers have been added to the human DNA, about 50% of them in the close gene vicinity and 22% in gene introns. For 60 such human-specific promoters, their activity was confirmed by *in vivo* assays, with the transcriptional level varying ~1000-fold from hardly detectable to as high as ~3% of β-actin transcript level. New polyadenylation signals have been provided to four human RNAs, and a number of potential antisense regulators of known human genes appeared due to human-specific retroviral insertional activity. This information is given here in the context of other major genomic changes underlining differences between human and chimpanzee DNAs. Finally, a comprehensive database, is available for download, of human-specific and polymorphic endogenous retroviruses is presented, which encompasses the data on their genomic localization, primary structure, encoded viral genes, human gene neighborhood, transcriptional activity, and methylation status.

The understanding of genetic basis, which accounts for obvious differences in phenotypes of humans and their closest relatives, chimpanzees, is one of the most interesting tasks of modern life sciences. This task is also challenging, sometimes puzzling, mostly due to strikingly high similarities in their genome structure and organization[1,2,3,4]. Indeed, an average divergence between human and chimpanzee DNAs is about 1.24%[5], being as low as only 0.5% in protein-coding regions[6]. Human and chimpanzee ancestor lineages diverged relatively recently in evolution, ~6 million years ago[7]. At present, we still do not know exactly what genetic traits make us humans, but a number of differences between human and great ape genomes have been identified. These changes explain, at least partly, some of the human peculiarities, although sometimes hypothetically. Generally, human-chimpanzee differences can be classified into four major groups:

1. Diverse chromosomal organization, including deletions, inversions, duplications, and translocations

2. Variations in copy number, genomic localization, and functional status among the pre-existing common sequences
3. Differences in protein coding regions
4. Lineage-specific genomic insertions of transposable elements (TEs)

TEs are DNA fragments, capable of self-reproducing and changing their location in the host genome, i.e., to transpose. These selfish repetitive elements proliferate either directly via their DNA copies (DNA transposons) or through RNA intermediates (retroelements), utilizing the mechanism termed "reverse transcription" and the RNA-dependant DNA polymerase enzyme, called reverse transcriptase (RT). The newly formed DNA copy of the element then integrates into the genome, using a combination of host and self-encoded proteins, depending on the TE origin[8]. Retroelements, which constitute >42% of human DNA, are the only class of TEs able to transpose in mammals[9,10]. Four retroelement families (L1, Alu, SVA, and HERV-K [HML-2]) were transpositionally active after the divergence of human and chimpanzee ancestries, thus forming a relatively modest fraction of human-specific inserts (~thousands of copies[11] compared to a total of ~3 million of human retroelements[12,13]).

Since 1975, when King and Wilson postulated — basing on the extreme similarity of human and chimpanzee genes and proteins — that a relatively small number of changes in genetic modules regulating gene expression could account for major phenotypic differences between these two specie[14], this conception has become classic. This review is devoted to human-specific endogenous retroviruses represented by only one family, HERV-K (HML-2), which are, probably, the best candidates for such a role of unique powerful gene expression regulatory modules, recently acquired by the human DNA.

### GENETIC DIFFERENCES BETWEEN HUMANS AND CHIMPANZEES

Millions of mutated loci, mostly single nucleotide polymorphisms (SNPs), are known to be polymorphic in humans. However, these latter human-specific records will not be considered here due to the lack of a functional role in human speciation for these sequences, which are presented only in a fraction of the human population. Human and chimpanzee genomes, both $\sim 3 \cdot 10^9$ bp in size, share ~98.8% sequence identity[5], thus making identification of functional human-specific sequences like finding the needle in a haystack. Theoretically, recent success in human and chimpanzee DNA sequencing projects[3,15] has provided an instrument for the direct comparison of genomes with the subsequent association of genomic changes with interspecies differences at the level of protein expression. However, in practice, the chimpanzee genome draft, currently available in public databases, is not sufficiently accurate for such a comprehensive study[16], although many successful large-scale bioinformatical screenings have been performed[17,18,19,20]. Plenty of lineage-specific substitutions, deletions, insertions, duplications, expressed pseudogenes, anonymous RNAs, and transposable elements have been identified nowadays, but the omnibus study still remains to be done.

#### Cytogenetic Differences

The comparison of human and African great ape karyotypes using fluorescent *in situ* hybridization has revealed the most important lineage-specific distinction, which is the fusion in the human lineage of two ancestral chromosomes (human chromosome 2), corresponding to chimpanzee chromosomes 12 and 13[21]. Other major findings are multiple pericentric inversions on human chromosomes 1 and 18[22,23], massive heterochromatin region rearrangments[23], extended interchromosomal DNA exchanges on the periphery of α-satellite DNA in centromeres[24], lack of AT-rich satellite in human subtelomeric regions[25], and, finally, lineage-specific rearrangements and amplifications of several gene families in nonrecombining parts of the Y chromosome[26]. Apart from translocations, insertions and deletions have together given rise to at least 150 Mb of the genomic DNA sequence that is either present or absent in humans as compared to chimpanzees, according to the recent estimation by Kehrer-Sawatzki and Cooper[27]. Interestingly, mostly chromosome ends were the "hot spots" of recent genome evolution[28].

### Emerging or Inactivation of Functional Genes

Few functional genes are known to distinguish human and ape DNA. The first example is the functional deletion of an exon within the protein-coding sequence of human gene *CMP* for syalic acid hydroxylase. Mutation, caused by the human-specific insertion of Alu retroelement into 92-bp-long *CMP* exon, disrupted normal open reading frame (ORF) for this enzyme and resulted in the lack of N-glycolyl neuraminic acid (Neu5Gc) on a surface of cell membranes[29,30]. Neu5Gc, thus, is replaced in humans by its precursor, N-acetyl neuraminic acid (Neu5Ac). This absense of Neu5Gc is the major biochemical distinction between human and chimpanzee, which, theoretically, may influence intercellular interactions and embryo development, e.g., brain organogenesis. Some other ancestor genes, mostly encoding olfactory receptors[31], have been lost or pseudogenized in the human lineage due to premature stop codon accumulations[20,32]. On the contrary, a transcribed human-specific sequence termed *c1orf37-dup* gene, encoding for short transmembrane protein of unknown function, is selectively expressed in several human tissues, including brain[33]. Also, a number of new copies of genes involved in immune response, such as leukocyte receptors or antigens, have been acquired, lost, or mutated during the recent human lineage evolution[34]. Finally, Pollard et al.[19] recently reported a novel human-specific gene, *HAR1F*, for a putative regulatory RNA that is expressed specifically in the developing human neocortex from 7 to 19 gestational weeks, a crucial period for cortical neuron specification and migration.

### Gene Duplications

Gene duplications may influence cell physiology by providing additional copies of transcribed genes, thus escaping the original qualitative control of gene expression. For example, seven to 11 copies of the olfactory receptor gene *OR-A* reside in human DNA, whereas the chimpanzee genome possesses only one copy of that gene. Different human copies are transcribed with different specificities, depending on their new genomic context[35,36]. Similarly, eight genes for keratinocyte growth factor (KGF) were mapped in human DNA, in contrast to only five copies in the chimpanzee[37].

### Lineage-Specific Nucleotide Substitutions

Millions of human-specific single nucleotide substitutions, short deletions, duplications, or microsatellite amplifications have been documented to date[4,27]. Many of them were mapped in regulatory gene regions or even in protein-coding sequences. For instance, the chimpanzee dopamin receptor gene *D4* has a 12-bp-long deletion, as compared with its human ortholog[38]. However, the biological significance of these numerous changes, accounting for a total of ~36 Mb in our DNA (mostly single nucleotide substitutions), is still waiting for a comprehensive analysis and systematization.

### Differences in Gene Expression

Identifying differentially transcribed sequences may be a better solution for the direct finding of functional genes that might be involved in human speciation[39]. For example, Nadezhdin et al. managed to identify differential transcription of a gene for transthyretin, the carrier of thyroid hormones, in the cerebella of humans and chimpanzees[40]. However, in practice, it is very difficult to obtain a sufficient umber of chimpanzee tissue samples for RNA isolation. Moreover, one has to compare tissues from the same physiological groups of donors, for instance, healthy aged females. Due to an extremely limited number of available chimpanzee tissue specimens, no statistically reliable comparison has been made so far, and the observed interspecies differences in gene expression remain frequently less in amplitude than the intraspecies ones[39].

### Transposable Elements

Four groups of retroelements remained transpositionally active after human-chimpanzee ancestral radiation and, consequently, created lineage-specific inserts. In this respect, the genome-wide bioinformatical recovery of lineage-specific TEs, recently performed by Devine and associates[11], provides an instrument for both quantitative and qualitative analysis of human-specific retroelement expansion. Of course, the figure of ~7800 identified elements is an underestimation of a real number of human-specific TEs, mostly due to the incomplete status of the chimpanzee genome draft. Moreover, in both human and chimpanzee genomes, primary structures of centromeric and peritelomeric heterochromatin regions, especially enriched in TEs, can be hardly established using the conventional shot-gun sequencing strategy.

The first group, presented by ~1200 human-specific members (which is significantly lower than previously predicted[41]), is the L1 family of authonomous retrotransposons. The full-length primate L1s are about 6 kb long, encoding two ORFs, for RT/integrase and RNA binding protein. However, L1 inserts are mostly 5'-truncated, gene expression–deficient copies that have been originated, probably, due to abortive reverse transcription[42]. The second and third groups, Alu (~300 bp long) and SVA (~1.5 kb in size) retroposons, are nonauthonomous TEs that recruit "heterologous" RT of the L1 origin for their own proliferation[43]. These two groups, presented in human DNA by ~5500 and ~860 lineage-specific copies, respectively, lack any protein-coding genes and can be regarded as parasites of L1 retrotranspositional machinery[9]. Finally, authonomous HERV-K (HML-2) endogenous retroviruses, harboring three functional and one regulatory gene(s), are the most complex human TEs. Other elements, identified by Mills et al.[11], were mostly pseudogenes mobilized by L1-encoded RT, or new satellite sequences. Together, human-specific retroelements constitute ~6.4 Mb of the human DNA (Fig. 1), which is sixfold lower than that formed by short nucleotide substitutions and 23-fold lower than human-specific deletions/duplications. However, such a modest proportion is somewhat compensated by the active role of functional genome reshapers that is being played by human retrotransposons[9,10,44,45,46]. TEs are known to be recombination hot spots (e.g., human-specific Alu-Alu recombinations resulted in deletion of at least 400 kb of human DNA[47]). It is known that retroelements like endogenous retroviruses, L1 and Alu, can modify the activity of pre-existing human genes, in particular by providing new promoters, polyadenylation signals, and additional splice sites[9,46,48,49]. Moreover, polymorphic L1s and Alu repeats have been shown recently to decrease transcription of the corresponding alleles when compared to the expression of retroelement-free alleles[50,51,52]. It should be noted here, that at least one-third of all human-specific retroelements has been mapped within or close to genes[11].

Therefore, TEs may well be one of the causative agents responsible for the phenotypic differences between *Homo sapiens* and its closest relatives, *Pan paniscus* and *P. troglodytes* chimpanzees. These differences can be envisioned to arise not from the appearance of any new and/or disappearance of old genes, but due to variations in the regulation of some genes common for the related specie. This review deals with human-specific endogenous retroviruses, which are probably the most interesting candidates for such a role due to their powerful regulatory elements, such as functional enhancers, promoters, splice sites, polyadenylation signals, and four viral genes involved, apart from retroviral life cycle, in immune suppression, antiviral protection, and cancer progression. In the present paper, I provide a detailed database for this group of human-specific TEs, including their genomic localization, distance from known genes, information about encoded proteins, and functional status, such as transcription and methylation in several human tissues.

### HUMAN-SPECIFIC ENDOGENOUS RETROVIRUSES: GENOMIC STRUCTURE

HERV-K (HML-2) is the sole group of endogenous retroviruses known to contain human-specific members. Group HERV-K (HML-2) occupies ~5% of the DNA created by insertions of human-specific TEs and is one of the best-studied families of human retroelements. At present, 134 human-specific members of this

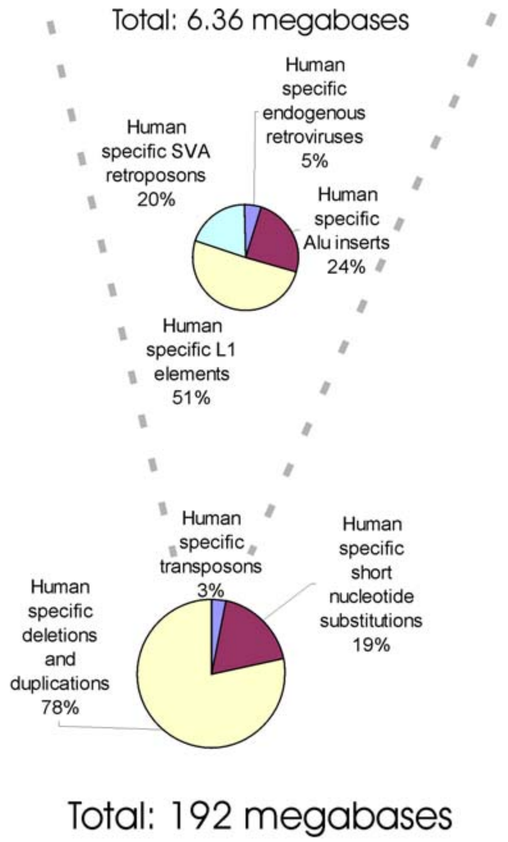

**FIGURE 1.** Endogenous retroviruses occupy ~5% of the DNA shaped by human-specific transposable elements, which, in turn, form only 3% of the total lineage-specific DNA.

family have been described elsewhere[53,54,55,56,57,58,59,60,61,62,63] that make up ~330 kb of the human DNA. Human-specific elements constitute a rather small fraction (~7%) of the HERV-K (HML-2) group, which is one of at least 40 distinct human endogenous retroviral (HERV) families[64]. In general, all HERVs are believed to be genomic traces of numerous germ-line retroviral infections[65] that occurred repeatedly during primate evolution[10,63]. This hypothesis was greatly supported by recent experiments on the artificial reconstruction of an active HERV-K (HML-2) progenitor[66]. The authors show that this element amplifies via an extracellular pathway involving reinfection, at variance with the non-LTR-retrotransposons (LINEs, SINEs) or LTR-retrotransposons, thus recapitulating *ex vivo* the molecular events responsible for its dissemination in the host genomes.

HERVs are composed of sequences related to retroviral *gag*, *prot*, *env*, and *pol* genes and flanked by ~1-kb-long so-called long terminal repeats (LTRs). LTR structure harbors functional enhancers[67], promoters, and polyadenylation signals[10] normally used for retroviral gene expression. However, it was recently demonstrated that LTRs may drive the transcription of adjacent host genomic sequences[68,69]. Most HERVs reside in the human genome as solitary LTRs, arisen most probably due to homologous recombinations between LTRs of full-length elements[70,71,72] (Fig. 2). However, some full-length

HERVs were reported to express viral genes in a variety of human tissues[73] and even to form virus-like particles in placenta[74,75].

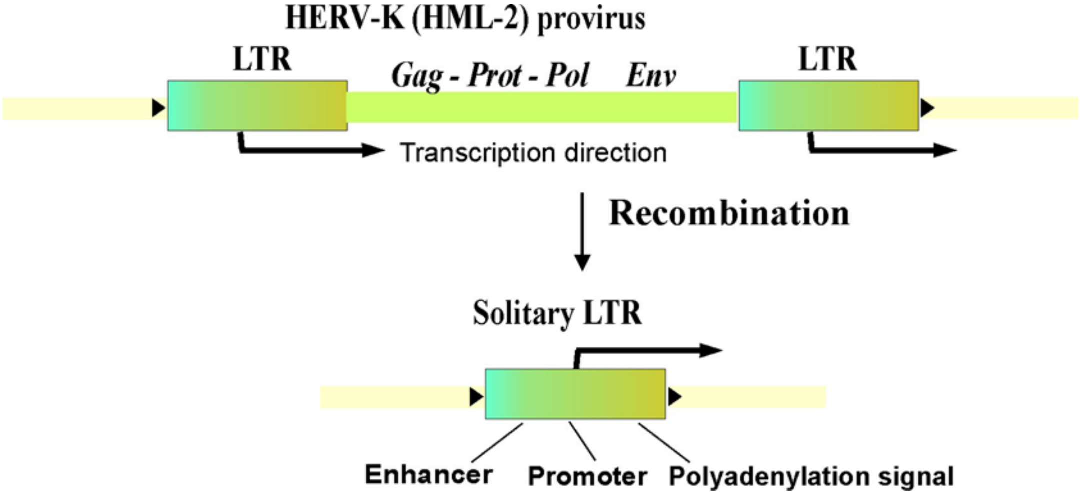

**FIGURE 2.** Major part of HERV-K (HML-2) elements exist in the form of solitary LTRs, arisen due to homologous recombination events between the identical LTRs flanking proviral genes *Gag*, *Prot*, *Pol*, and *Env*. Each LTR harbors polyadenylation signal, enhancer, and promoter elements, and can initiate transcription of the downstream genomic loci.

Similarly to other HERVs, the HERV-K (HML-2) group comprises mostly transpositionally deficient and transcriptionally silent representatives[76]. However, some family members (mostly human-specific entries) not only retained their transcriptional activity[77], but also probably still possess some infectious potential[62,78], thus making this group the most biologically active endogenous retroviral family of the human genome[72,79,80]. This peculiarity of the HERV-K (HML-2) group is, most probably, due to its exclusive, rather recent activity in human evolution[60,78,81]. Indeed, recent integrants have more intact ORFs and less mutated regulatory sequences (mostly nuclear factor binding sites, frequently enriched in CpG dinucleotides) as compared to ancient inserts[82]. The HERV-K (HML-2) group comprises at least 55 full-length members (termed proviruses)[63] and ~2000 solitary (solo) LTRs[60].

Having a variety of potential regulatory sequences, such as promoter, enhancer, transcriptional factor binding elements, splice site, and polyadenylation signal, HERV-K (HML-2) LTRs are generally believed to possess the full transcriptional regulatory potential of endogenous retroviruses. Accordingly, it was demonstrated that solitary HERV-K LTRs specifically bind host cell nuclear proteins[83,84], serve as tissue-specific transcriptional promoters and enhancers in transient transfection experiments[67,85,86], are differentially methylated in various human cell types[85,87,88], and, finally, are transcribed *in vivo* in many tissues[77,89]. In addition, it was hypothesized that HERV-K LTRs may contribute to the host gene regulation network by acting in *cis* (by providing regulatory elements) or in *trans* (by driving expression of antisense transcripts)[9,71,90,91]. Interestingly, Romano and colleagues recently demonstrated that LTR sequences had a higher substitution rate than the rest of the genome[63]. This higher mutation rate could underline LTR regulatory potential, as a higher rate of nucleotide substitution in the LTR could lead to its inactivation, counteracting its deleterious effects[63].

It should be noted here that essentially all human-specific endogenous retroviruses have been identified a few years before having the chimpanzee genome sequence draft published. To this end, a combination of experimental and bioinformatical methods was used. First, the presence/absense of the analyzing TE insert in the genomes under study can be screened using the simple PCR test called "locus-specific PCR". Genomic DNAs are amplified with primer pairs designed to the corresponding unique TE-flanking regions in the human genome (Fig. 3). An amplicon with the TE insert is supposed to be longer than without it. For instance, as depicted in Fig. 3, a ~1-kb-longer amplicon suggests the presence of the HERV-K (HML-2) solo LTR insert in the human genome and its absence in the chimpanzee DNA. Similar tests are employed to investigate intraspecies variations of the TE content as well (e.g., for finding TE polymorphisms in population genetics studies)[92,93,94,95,96]. Alternatively, lineage-specific inserts

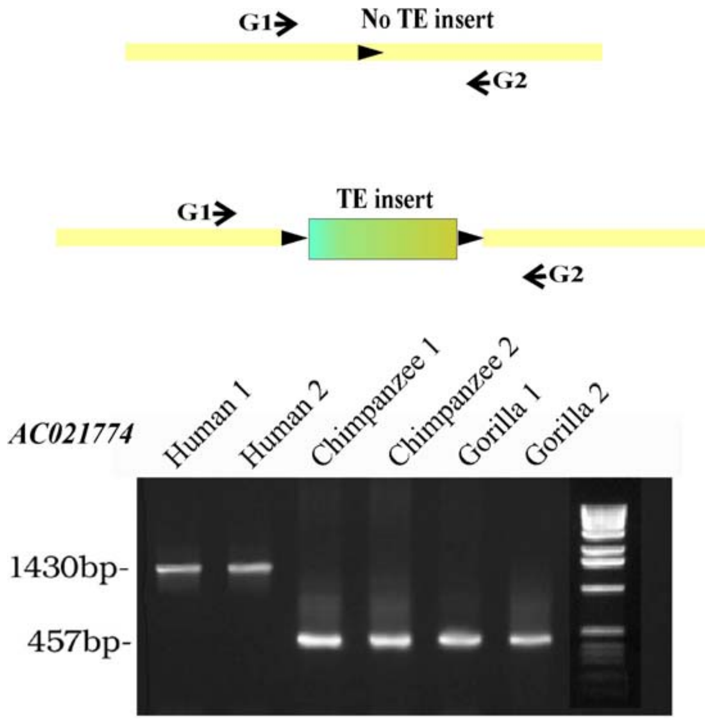

**FIGURE 3.** Schematic representation of the locus-specific PCR. Orthologous loci, which lack the TE insert, give shorter PCR fragments when amplified with TE-flanking unique genomic primers. This example illustrates locus-specific PCR of orthologous human and great ape genomic loci with primers, which flank the human HERV-K (HML-2) solitary LTR from genomic contig AC021774. Human amplification products are ~1 kb greater in size than the amplified loci from chimpanzee and gorilla DNAs, which suggests the presence of ~1-kb-long LTR in human genome and its absence from the apes' orthologous loci.

can also be identified by more direct approaches, such as genomic subtractive hybridization of TE-containing loci[81,97], microarray hybridization of TE-flanking DNA[57], or by the modified differential display[89,98].

### HS Family

The above strategies enabled the identification of 41 human-specific integrations of HERV-K (HML-2) proviruses and solo LTRs[53,54,57,58,60,61,62]. Interestingly, human-specific retroviral LTRs appeared to share a significant sequence identity[56,60]. Medstrand and Mager[60] demonstrated close structural similarity of eight human-specifically integrated HERV-K (HML-2) LTRs revealed by them. Having analyzed a number of randomly chosen LTRs, the authors noted that the nucleotide sequences of the human-specific entries were highly identical and formed a separate cluster on a phylogenetic tree. They also showed a parallel amplification of sequences similar to the LTRs from that cluster in the genomes of human, chimpanzee, and gorilla. In contrast, HERV-K (HML-2) proviral genes were too conservative to display any lineage-specific features. More recently, basing on the structures of 41 established human-specific integrants, we derived a consensus sequence for a fraction of recently inserted LTRs (termed HS

LTRs). These LTRs were very similar, with the values of intragroup divergence varying from 0.1 to 3.5% with the average of 2.3%, whereas the only exception was the human-specific element *AC022567* that was rather different from other LTRs (6% average divergence) and could not be assigned to that group[56].

The deduced HS consensus sequence has eight unique diagnostic nucleotide positions absent from other LTR group consensus sequences[56]. This consensus structure was further used to identify new HS family members in human genome databases. A total of ~150 HS elements have been identified with the average intragroup divergence of 2.3%.

A detailed sequence analysis of the HS family allowed us to further subdivide it into two subfamilies termed HS-a and HS-b. The HS-a subfamily, which is highly identical to the HS consensus sequence, is characterized by intragroup divergence of 1.5%, which corresponds to the evolutionary age of ~5.8 million years (Myr), assuming the mutation rate of HERV-K (HML-2) LTRs to be 0.13%/1 Myr[10]. In line with this estimation, all HS-a LTRs appeared to be human specific. On the contrary, the HS-b subfamily, which is known to include some members common for human and chimpanzee DNAs, is evolutionarily older, with the intragroup divergence of 2.6% and the age of 10.3 Myr. It is noteworthy that 86% of all HS proviruses harbor HS-a LTRs vs. only 14% of them having HS-b LTRs. Moreover, 13% of HS-a LTRs are incorporated in proviruses vs. only 4% of older HS-b LTRs. This represents an obvious example of temporal inactivation of an evolutionary older group of endogenous retroviruses.

Overall, about 86% of all HS elements have been shown to be human specific. The peak retroposition activity of this group runs back to the period after the divergence of the human and chimpanzee ancestor lineages that occurred 4–6 Myr ago[10,99]. At least 12 HS family members are even polymorphic in the modern human population[62,78,99,100], thus suggesting that this family remained transpositionally active up to recent times in the evolutionary history of *Homo sapiens*, being probably still active at present[62,79,101,102]. In all likelihood, the progenitor HS family sequences emerged in the genome of the common ancestor of the gorilla, chimpanzee, and human lineages about 10.7 Myr ago, having given rise to the HS-b group. This group, being retropositionally active 5.8 Myr ago, i.e., roughly at the time of the ancestor human and chimpanzee lineages divergence, has in turn generated the retropositionally more active HS-a group, which currently represents a major part (>60%) of the whole HS family. The HS-b group remained active after the human-chimpanzee divergence, both in the human and chimpanzee lineages[56]. Interestingly, the five linked nucleotide substitutions underlying the difference between the HS-a and HS-b groups lie in the region previously shown to be a *cis*-negative regulator of an HS-b LTR promoter (GenBank Accession No. *L47334*). Deletion of 70 bp from this region results in a twofold increase in the promoter activity[86]. Theoretically, a higher retroposition rate of the evolutionarily younger HS-a group might result from these five mutations in the LTR-negative regulator region.

The representatives of both the HS-a and HS-b groups were retropositionally active up to relatively recent times in the human evolution. This is supported by the finding of HS elements polymorphic in humans, e.g., HS-b solitary LTR (*Z80898*)[60] and HS-a members proviruses HERV-K 113 (*AY037928*) and HERV-K 115 (*AY037929*)[62]. The identification of the human-specific LTR that cannot be assigned to the HS family (*AC022567*; see above) suggests that at least three HERV-K (HML-2) LTR "master genes" (HS-a, HS-b, *AC022567*) were active in the hominid lineage. The HS family, whose members retained their transcriptional activity[77,80,103,104] and were found to be tissue-specifically methylated[85,87,88], is thought to be the most biologically active retroviral family in human cells.

Among 134 human-specific endogenous retroviral inserts, 17 are full-size proviruses, four and three are, respectively, 5'- and 3'-truncated elements, and 110 elements are solitary LTRs (Supplementary Table 1). Twelve human-specific endogenous retroviral inserts (9%) are polymorphic in the human population, thus suggesting their very recent integrations (Supplementary Table 1). Theoretically, some full-length elements, which have intact or almost intact ORFs (see below), could preserve their transpositional activity and infectious potential until now[82]. Finally, a possibility exists that polymorphic proviral inserts may be associated with human diseases[105].

### VIRAL LIFE CYCLE

Similarly to other retroelements, the life cycle of HERV-K (HML-2) endogenous retroviruses comprises reverse transcription of viral genomic RNA, followed by the integration of a nascent DNA copy into genomic DNA of the host cell[66,106]. Importantly, retroviral genomic RNA differs from genomic copy by the absence of LTRs, which are built during the reverse transcription, a multistep complex process including several template-switching events[107], described in detail elsewhere (e.g., [108,109]). The newly inserted element is usually flanked by short, few-basepair-long, tandem repeats of genomic DNA from HERV preintegrational site. However, significantly longer repeats have been documented for few individual HERV-K (HML-2) entries[110].

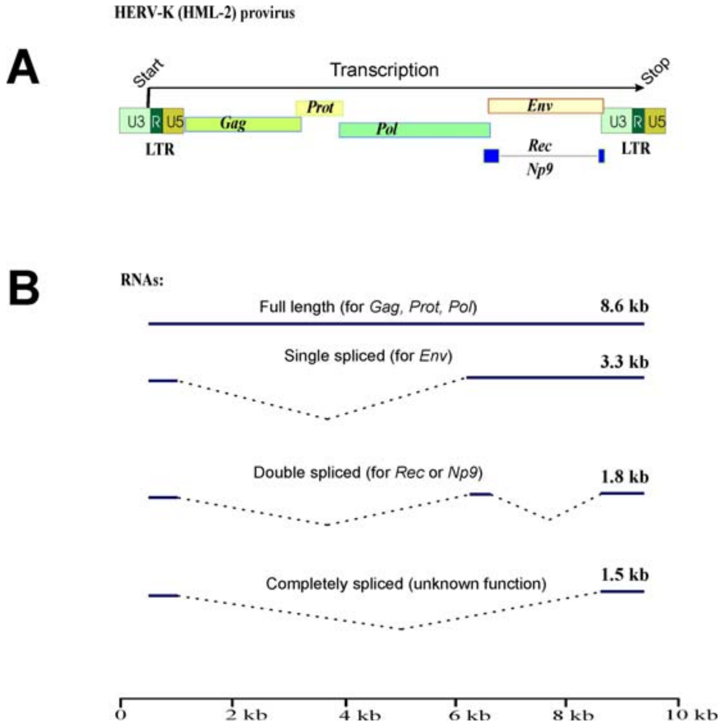

**FIGURE 4.** Functional genes, encoded by HERV-K (HML-2) endogenous retroviruses. (A) Genomic organization of the reconstituted full-size functional provirus. Apart from "classical" retroviral genes *Gag*, *Prot*, *Pol*, and *Env*, an additional gene termed "*Rec*" or "*Np9*", depending on the retroviral type, is encoded. (B) Different types of proviral transcripts. Full-length subgenomic transcript encodes for Gag-Prot-Pol polyprotein, single-spliced product codes Env protein, double-spliced RNA is for Rec/Np9, whereas ~1.5-kb-long completely spliced transcript of unknown function appears to lack any functional ORFs.

The inserted proviral copy is normally transcribed from its functional promoter on the U3 region of the 5' LTR (Fig. 4A). Transcription stops at the 3'-terminal LTR by using U5-located polyadenylation signal AATAAA. The polyadenylated full-length transcript can be further spliced, thus giving at least three different splice forms (Fig. 4B). Unspliced transcript encodes for 160-kDa viral polyprotein Gag-Prot-Pol. Two –1 ribosome frameshifts are needed to translate the Gag-Prot-Pol polyprotein[82]. The precursor polyprotein is then processed by the Prot (retroviral protease) intramolecular activity, and the mature proteins are released. The Gag protein is further cleaved to release matrix, core, and nucleocapsid proteins[82,111]. Pol is the retroviral RT, possessing RNase H activity. Importantly, HERV-K (HML-2) Prot has an additional dUTPase domain that protects against toxic misincorporation of dUTP into cDNA during reverse transcription. The spliced transcript encodes for the envelope protein (Env) that is needed to infect human cells via a cellular receptor[82].

Double-spliced RNA codes a short regulatory protein. Among human-specific HERV-K (HML-2) elements, two types of proviruses exist. Unlike type 2 proviruses, type 1 elements share a 292-nt deletion in the *env* region. Apart from fusion of *pol* and *env* genes, this deletion also gives rise to a difference between the two isoforms of regulatory proteins encoded by the double-spliced transcripts. Type 2 proviral transcripts, 1.8 kb long, code the 15-kDa accessory protein Rec (alternavely called cORF[112]), which is the only known auxillary factor encoded by HERVs[82]. It has a striking functional homology to lentiviral RNA-binding nuclear export proteins like the HIV and HTLV proteins Rev and Rex[82]. Similarly to those proteins, Rec binds to unspliced or partially spliced viral transcripts and mediates their transfer to the cytoplasm, where they escape the splicing machinery and can be translated[113]. The Rec binding site, termed Rec Responsive Element, is a highly structured RNA motif within the U3R region of the 3' LTR. Interestingly, this functional motif can be recognized by the HTLV Rex protein that can at least partly substitute for the Rec function[82,113,114]. Rec is specifically accumulated in the nucleoli, thus suggesting a role for them in the HERV life cycle. Type 1–specific double-spliced RNA product, called Np9, is a 9-kDa protein that shares only the N-terminal 15 aa residues with Rec[82,115,116]. Similarly to Rec, Np9 accumulates in the nucleus. Although Np9 expression has been documented in many tissues and cell lines, the exact molecular function for this protein remains unclear.

Finally, ~1.5-kb-long completely spliced proviral transcripts (Fig. 4B) appear to lack any protein-coding regions and may have only some regulatory functions (if any). The study of tight regulation of provirus transcription, splicing, and protein expression is under way in many laboratories now. The overall observation is that proviruses are expressed at much higher levels in germ-line cells, especially in germ cell tumors[117,118].

### EXPRESSION OF VIRAL PROTEINS

The bioinformatical analysis of human-specific proviruses revealed that they may harbor a total of 11 functional genes for Gag, 12 for Prot, nine for Pol, eight for Env, and nine for Rec (Supplementary Table 1). Except for their functions in the viral life cycle, multiple physiological roles of these genes have been reported for human cells[82]. First, HERV-K (HML-2) proteins are actively expressed in a variety of human tissues[82]. Indeed, antibodies against multiple HERV-K proviral Env epitopes were found in 30% of healthy individuals[119]. Interestingly, in line with the enhanced expression of HERV-K in germ cell tumors, a significant increase in frequency and titer of antibodies against proviral proteins in patients suffering from testicular cancers has been documented (60% against 4% in healthy control group)[111]. Importantly, shortly after the elimination of the tumor, the antibody titers dropped and became undetectable 5 years after surgery[111]. Apart from expression in other tumors[120,121], increased proviral protein production was also detected in placentas and in embryonic tissues, in line with the identification of putative responsive elements for several pregnancy hormones within the HERV-K LTRs[122,123]. Theoretically, the expression of proviral proteins may trigger autoimmune diseases, but no direct proofs have been provided for this concept, except increased proviral transcript levels[103] and finding anti-HERV protein antibodies in sera from several groups of patients suffering from these malignancies[82].

Gag protein expression may induce massive T-cell stimulation or apoptosis[124]. Endogenous Prot genes may help exogenous retroviruses, such as lentiviruses, to infect the host cells. The primate lentiviruses HIV and simian immunodeficiency virus (SIV) do not express their own dUTPase, and it is believed that a host cell–endogenous retroviral enzyme (Prot) provides this activity during reverse transcription[125,126,127], in line with the recent observations that HIV-1 infection may increase the expression of HERV-K (HML-2) proviruses *in vitro*[128] and *in vivo*[129,130]. Rec and Np9 activities may interfere with normal nuclear cytoplasmic transport mechanisms[131] or even serve as inducers of organ-specific tumorogenesis[102].

Finally, the Env protein has an immunosuppressive domain that inhibits T- and B-cell activation[132]. The latter peculiarity might be related to an increased HERV-K expression in some tumors[82,133]. Besides deleterial effects, endogenous Env production can provide to the host cell a partial resistance to infection of pathogenic exogenous counterparts or related retroviruses by receptor interference[134,135,136], as this is the case for endogenous Jaagsiekte sheep retrovirus (JSRV) that blocks the entry of the corresponding exogenous virus. Both forms use hyaluronidase-2 as a receptor for entry, implying interference between endogenous JSRV Env and exogenous viruses[135]. Endogenous Gag protein might also be involved in antiviral host cell protection. For instance, the expression of murine endogenous *gag*-sequence Fv1 blocks certain strains of mouse leukemia virus (MLV) soon after entry[137], most probably, due to a direct encounter with the incoming viral capsid[82].

### REGULATORY POTENTIAL OF ENDOGENOUS RETROVIRUSES

Not only proteins, but also viral regulatory sequences contributed to the evolution of human genome and transcriptome[73,138,139]. At least hypothetically, 134 human-specific HERV-K (HML-2) LTRs having functional enhancers, promoters, polyadenylation signals, and splice sites could be involved in the following five pathways of human transcriptional regulation (Fig. 5):

1. LTR enhancer activity may change transcriptional profiles of the pre-existing neighboring genes.
2. Promoters provided by the LTRs may drive transcription of downstream genomic sequences, thus creating new genes.
3. LTR polyadenylation sites may cause abnormal termination of the read-through transcripts.
4. LTR splice sites can alter exon-intronic structure of the original genes.
5. LTRs may regulate expression of the host genes via RNA interference mechanisms.

Below I have tried to put together all the experimental material available to date that can properly address these items. Many above pathways deal with the regulation of expression of the pre-existing human genes. In this respect, it is important that 61 human-specific LTRs (45% of the total number of lineage-specific elements) are located within or close to known human genes (Supplementary Table 1).

#### LTR Enhancer Activity

HERV LTR enhancer activity was extensively studied *in vitro*, mostly not for human-specific members[67,140,141], with the only exception of the solitary LTR from genomic contig L47334 that is restricted to human DNA[67]. In transient transfection experiments on a panel of 10 mammalian cell lines, this LTR has demonstrated enhancer activity only in Tera-1 human testicular embryonal carcinoma cells (thus showing an approximately eightfold increase in luciferase expression, as compared to control plasmid lacking the enhancer element tested). This finding clearly suggests that, at least theoretically, human-specific LTRs may be involved in a specific activation of neighboring functional genes, especially in embryonic and cancer tissues[67].

#### Promoter Activity Human-Specific LTRs

Promoter activity of human-specific LTRs was investigated in both *in vitro* and *in vivo* assays. In transient transfection experiments with the luciferase or GFP reporter genes, the same human-specific element from contig L47334 displayed very low promoter activity in three of the 10 cell lines tested, moderate activity (10–20% of the SV40 early promoter) was observed in six cell lines and, finally, the maximal value of ~100% of SV40 promoter activity was obtained in Tera-1 cells, similarly to the above enhancer activity tests[86]. In the experiments by Lavie et al.[85], five human-specific proviral 5' LTRs demonstrated the promoter strengths as high as 5–15% of the cytomegalovirus (CMV) promoter activity

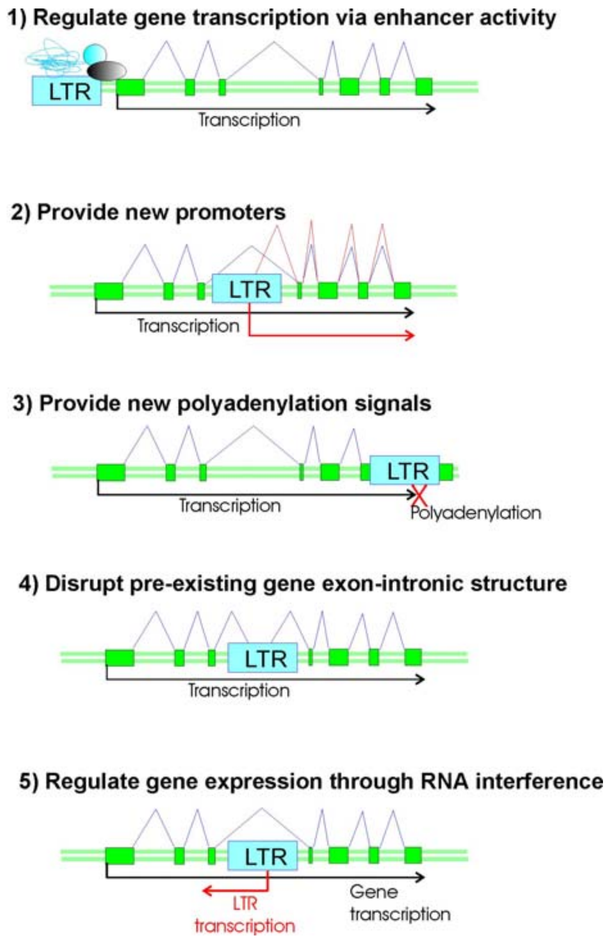

**FIGURE 5.** Five potential mechanisms that comprise the recruitment of human-specific endogenous retroviruses for modulating activity of pre-existing genes. At present, direct experimental evidence has been provided for the *in vivo* enhancer, promoter, and transcription termination activities of the human-specific endogenous retroviruses (see text).

in Tera-1 cells (AP000776, 15% of CMV promoter expression; AC025757, 9%; AC072054, 8%; AC025420, 6%; and AL590785, 5%). The authors demonstrated that the promoter activities of these elements directly depend on the methylation status of their CpG dinucleotides. Interestingly, the same five LTRs were strongly transcriptionally repressed in T47D cells[85].

In *in vivo* experiments, 5' RACE (rapid amplification of cDNA ends)-based mapping of transcriptional start sites for five actively transcribed human-specific LTRs provided evidence for the presence of two functional promoter regions within the LTR sequence[68]. Both promoters possess TATA box motif and other upstream regulatory sequences. The first promoter was the canonical element located in the LTR U3 region, whereas the second one was mapped in the very 3' terminus of the LTR R region. Both promoters appeared to be active in solitary LTRs and in full-length proviruses. Surprisingly, this second noncanonical element was even more active than the classical U3-located retroviral promoter. Therefore, the R region is excluded from most transcripts initiated on LTRs, whereas a classical retroviral life cycle model implies that the transcription is driven from between the LTR U3 and R elements (first promoter), and the R transcript is a 5'-terminal component of the newly synthesized proviral RNA. Such a mode of proviral DNA transcription is a basis of the life cycle that provides the possibility of template jumps during proviral RNA reverse transcription. A shift of the transcriptional start site can be explained by the presence of at least two alternative promoters within the LTR, one of which is normally used for viral gene expression, and the other for transcription of retrotransposition-competent copies of the integrated provirus. The latter type of transcripts is supposed to be far less abundant, which basically corresponds to the above observations. It should be mentioned that alternative promoters with unknown functions were found earlier for many other retrotransposons[9,46,142].

The comprehensive study of the expression of human-specific LTRs *in vivo* in human germ-line tissue (testicular parenchyma) and in the corresponding tumor (seminoma) sampled from the same patient was recently performed[58]. These were chosen because of markedly high endogenous retroviral transcriptional activity in germ-line cells, which is most probably needed to make *de novo* retroviral integrations inheritable[76,143]. To this end, a new experimental technique that makes it possible to detect repetitive element promoter activity has been developed[55]. This technique, termed GREM (genomic repeat expression monitor), combines the advantages of 5'-RACE and nucleic acid hybridization techniques. GREM is based on hybridization of total pools of cDNA 5' terminal parts to genome wide pools of repetitive elements flanking DNA, followed by selective PCR amplification of the resulting hybrid cDNA-genome duplexes. A library of cDNA/genomic DNA hybrid molecules obtained in such a way can be used as a set of tags for individual transcriptionally active repetitive elements[55]. The method is both quantitative and qualitative, as the number of tags is proportional to the content of mRNA driven from the corresponding promoter active repetitive element. The GREM outcome was a set of amplified cDNA/genomic DNA heteroduplexes, below referred to as Expressed LTR Tags (ELTs), which were further cloned and sequenced.

To my knowledge, this study was the first quantitative and qualitative comprehensive characteristic of functional promoters provided by a particular group of genomic repetitive elements. The data obtained in such a way suggest that at least 45% of human-specific LTRs possessed promoter activity, and a total of 60 new human promoters have been identified. Individual LTRs were expressed at markedly different levels ranging from ~0.001 to ~3% of the housekeeping β-actin gene transcript level. Although HS elements formed several subclusters on a phylogenetic tree[56,60], no clear correlation between LTR primary structure and transcriptional activity was found. In contrast, the LTR status (solitary, 5' or 3' proviral) was an important factor affecting LTR activity; promoter strengths of solitary and 3' proviral LTRs were almost identical in both tissues, whereas 5' proviral LTRs displayed higher promoter activity (approximately two- and fivefold greater in testicular parenchyma and seminoma, respectively). These data suggest that a proviral sequence harbors some yet unknown downstream regulatory elements that provide significantly higher 5' LTR expression, especially in seminoma. Another important factor affecting promoter activity was the LTR distance from genes; the relative content of promoter-active LTRs in gene-rich regions was significantly higher than in gene-poor genomic loci (Supplementary Table 1).

The data obtained also suggest a selective suppression of transcription in both tissues for proviral 3' LTRs located in gene introns. Such a transcriptional suppression might be aimed at silencing of the proviral gene expression in gene-rich regions. In testicular parenchyma, the promoter strength of intronically located solitary LTRs was also significantly decreased. This may suggest yet unknown mechanism(s) for selective suppression of "extra" promoters generated due to mutations or viral integrations and located within gene introns or very closely to genes. Such a mechanism might minimize possible destructive effects of undesirable transcription. Many transcriptionally competent LTRs were mapped near known human genes, and as many as 86–90% of all genes located in close proximity to promoter-active LTRs are known to be transcribed in testis. However, in general no clear-cut correlation was observed between transcriptional activities of genes and closely located LTRs[58]. Overall, LTRs provided at least 60 functional human-specific promoters for host nonrepetitive DNA that are transcribed at different levels ranging from ~0.001 to ~3% of β-actin transcript level.

### LTR Polyadenylation Signals

Inspection of human transcript databases (the present study) revealed four different RNAs that terminate at human-specific LTRs (elements 126, 128, 129, 139 [Supplementary Table 1]). Among these transcripts, three RNAs lack any ORFs, whereas one (BC092439) encodes for a 8-kDa protein of unknown function, highly similar to human protein GON4L, a transcription factor that may function in cell cycle control[144]. Therefore, at least one polyadenylation signal provided by human-specific LTR is recruited for transcriptional termination of a functional protein-coding RNA.

### LTR Splice Sites

No read-through transcripts using human-specific endogenous retroviral splice sites were found. Note that among 29 human-specific LTRs located in introns of known genes, 28 elements (or 97%) are in the opposite orientation relatively to gene transcription direction. This most probably occurred due to a selective loss of alleles having LTRs in gene introns in the positive orientation because of LTR functional polyadenylation signal and splice sites[45,145,146].

### LTRs as the Antisense Regulators

Among 28 human-specific LTRs located in gene introns in the antisense orientation relatively to gene transcription direction (Supplementary Table 1), 15 elements have been shown to be promoter active in human germ-line cells[58]. High expression levels of certain intronic LTRs might suggest the possibility of their involvement in antisense regulation of pre-existing genes[146]. This regulatory mechanism is based on formation of the double-stranded RNA between mRNA and the antisense transcript, followed by catalytic degradation of RNAs containing the sites homologous to the double-stranded fragment[90]. For example, human-specific LTR 91 (Supplementary Table 1) can be a tissue-specific regulator (e.g., an enhancer activating the cryptic promoter) of expression of two human transcripts (GenBank accession numbers AA704979 and R99122), complementary to the second exon of the gene *CEBZ* for the transcriptional regulator CCAAT-binding factor, which determines transcription from the hsp70 promoter[147]. Both transcripts can be regarded as possible antisense regulators of *CEBZ*. A bias in the expression profile of *CEBZ* could result in numerous effects on the transcription of other genes. However, more direct experimental data are needed to assess the impact of human-specific LTRs in the antisense regulation of human genes in a comprehensive and unambiguous way.

## DATABASE OF HUMAN-SPECIFIC ENDOGENOUS RETROVIRUSES

This database is an updated improved version of the previously published expressed human HERV database[58]. Supplementary Table 1 is the detailed database, which encompasses all human-specific endogenous retroviral inserts identified to date (a total of 134 elements). Some of these retroviral sequences were previously annotated in three other datasets: in the database of human retrotransposon insertion polymorphisms[148], in the HERVd database of human endogenous retroviruses[149], and in the database of human-specific transposable elements[11]. However, many human-specific elements are unique to the present database.

In the first column, retroviral IDs are given. In the second (column A), GenBank accession numbers of genomic contigs, including the respective element, are provided. Information on the element orientation and coordinates within the reference contig (typed in blue) are given in cell comments. HERVs polymorphic in the human population are marked by a light green background (12 elements).

Column B contains information about HERV mapping using the UCSC genome browser (http://genome.ucsc.edu/cgi-bin/hgGateway). Comments to each cell indicates detailed sequence information and chromosomal coordinates.

Column C — distances (D) of human-specific elements from known genes or mapped complete cDNAs. Plus and minus mean the presence or the absence of closely located genes, respectively. HERVs of group 1 (shown in white), D > 35 kb, 69 elements; group 2 (shown in light green), 5 ≤ D ≤ 35 kb, 20 elements; group 3 (shown in green), HERVs located within gene introns or at D < 5 kb, 35 representatives; elements from group 4 (shown in dark green) were found within exons of known non-LTR–promoted human cDNAs, thus partly or wholly read-through transcribed, 10 representatives.

Column D — status of human-specific elements (solitary LTR, provirus); structural features (if any) are described in cell comments.

Column E — information about putative ORFs encoded by the corresponding proviruses. In comments, the deduced amino acid sequence data for the respective proteins are given.

Column F represents data on methylation of individual elements, recently reported for several human tissues[85,87,88]. Relatively strongly methylated elements are shown in white, strongly demethylated in dark aquamarine. For intermediate methylation statuses, mid colors are used. More detailed information is given in cell comments.

Column G — RT-PCR data on the corresponding individual LTR transcription (in cell comments), obtained elsewhere[55,58,77,85,89].

Finally (column H) — expressed LTR tag (ELT, see above) frequencies for individual LTRs, in normal testicular parenchyma (Par) and in seminoma (Sem). ELT frequencies were calculated as a ratio of the number of the ELTs to the total number of ELTs for each tissue.

To my knowledge, this database is the first attempt to characterize in detail a particular group of human-specific repetitive elements. The information provided therewith might be valuable to those interested in the comprehensive recovery of functional genomic differences between human and chimpanzee.

### CONCLUDING REMARKS

In this review, a small particular family of human-specific repetitive elements has been considered. Although modest in terms of its copy number, this group appeared to modify the human genome activity by endogenizing ~50 functional copies of viral genes that may have important implications in the immune response, cancer progression, and antiretroviral host defense. A total of 134 potential promoters and enhancers have been added to the human DNA, about 50% of them in the close gene vicinity and 22% in gene introns. For 60 such human-specific promoters, their activity was confirmed by *in vivo* assays, with the transcriptional level varying ~1000-fold from hardly detectable to as high as ~3% of β-actin transcript level[58]. New polyadenylation signals have been provided to four human RNAs and a number of potential antisense regulators of known human genes appeared due to human-specific retroviral insertional activity. At present, we do not know whether these changes were evolutionarily significant for human-chimpanzee lineage divergence; however, further experimental functional tests of human-specific endogenous retroviruses will definitely add clarity to the current understanding of genetic traits that make us humans.

### ACKNOWLEDGMENTS

The author was supported by the Molecular and Cellular Biology Program of the Presidium of the Russian Academy of Sciences, by the grant MK-4227.2007.4 of the President of the Russian Federation and by the grants 05-04-50770-a and 05-04-48682-a from the Russian Foundation for Basic Research.
